# Surveillance of Antimicrobial Susceptibility of Anaerobe Clinical Isolates in Southeast Austria: *Bacteroides fragilis* Group Is on the Fast Track to Resistance

**DOI:** 10.3390/antibiotics10050479

**Published:** 2021-04-21

**Authors:** Elisabeth König, Hans P. Ziegler, Julia Tribus, Andrea J. Grisold, Gebhard Feierl, Eva Leitner

**Affiliations:** 1 Diagnostic & Research Institute of Hygiene, Microbiology and Environmental Medicine, Medical University of Graz, A 8010 Graz, Austria; elisabeth.ullrich@medunigraz.at (E.K.); hans.ziegler@medunigraz.at (H.P.Z.); tribus.julia@gmail.com (J.T.); andrea.grisold@medunigraz.at (A.J.G.); gebhard.feierl@medunigraz.at (G.F.); 2 Section of Infectious Diseases and Tropical Medicine, Department of Internal Medicine, Medical University of Graz, A 8010 Graz, Austria

**Keywords:** nosocomial infections, anaerobic bacteria, antimicrobial susceptibility testing, *B. fragilis*-group

## Abstract

Anaerobic bacteria play an important role in human infections. *Bacteroides* spp. are some of the 15 most common pathogens causing nosocomial infections. We present antimicrobial susceptibility testing (AST) results of 114 Gram-positive anaerobic isolates and 110 *Bacteroides-fragilis*-group-isolates (BFGI). Resistance profiles were determined by MIC gradient testing. Furthermore, we performed disk diffusion testing of BFGI and compared the results of the two methods. Within Gram-positive anaerobes, the highest resistance rates were found for clindamycin and moxifloxacin (21.9% and 16.7%, respectively), and resistance for beta-lactams and metronidazole was low (<1%). For BFGI, the highest resistance rates were also detected for clindamycin and moxifloxacin (50.9% and 36.4%, respectively). Resistance rates for piperacillin/tazobactam and amoxicillin/clavulanic acid were 10% and 7.3%, respectively. Two *B. fragilis* isolates were classified as multi-drug-resistant (MDR), with resistance against all tested beta-lactam antibiotics. The comparative study of 109 BFGI resulted in 130 discrepancies in 763 readings (17%) with a high number of Very Major Errors (VME) and Major Errors (ME). In summary, resistance rates, with the exception of clindamycin and moxifloxacin, are still low, but we are facing increasing resistance rates for BFGI. Surveillance studies on a regular basis are still recommended.

## 1. Introduction

Anaerobic bacteria play an important role in a variety of human infections. Among anaerobic bacteria, the *Bacteroides fragilis* group represents one of the most important anaerobic clinical pathogens and ranges under the 15 most common pathogens causing nosocomial infections [1,2]. Different Gram-positive anaerobic cocci like *Finegoldia magna, Peptostreptococcus anaerobius,* and *Parvimonas micra* account for approximately 25–30% of all isolated anaerobic bacteria from clinical specimens [3]. Within the Gram-positive anaerobe rods, *Cutibacterium acnes* plays an important role, especially in prosthetic joint infections [4]. Monomicrobial and polymicrobial anaerobic infections are usually treated empirically, based on surveillance reports of the susceptibility patterns of these pathogens. Routine susceptibility testing of anaerobic bacteria is not generally recommended for polymicrobial infections, but should be considered for specific clinical situations and in monomicrobial infections [5,6]. For empiric therapy strategies, resistance-surveillance is recommended due to an increase in resistance, especially in *Bacteroides fragilis* group isolates (BFGI) [1,7,8]. Nevertheless, cultivation and isolation of anaerobes is time-consuming, especially when susceptibility testing is required. The gold standard of susceptibility testing by agar-dilution method is not suitable for a routine diagnostic laboratory. Other techniques like the MIC gradient testing method (e.g., ETEST^®^) are costly and not always affordable for smaller laboratories or laboratories of middle- and low-income countries. As studies on the antimicrobial susceptibility profile of anaerobic bacteria are underrepresented in the literature [9], we present antimicrobial susceptibility testing (AST) results of 114 Gram positive anaerobic isolates and 110 BFGI determined by MIC gradient testing derived from clinical specimens in Southeast Austria. Furthermore, we performed disk diffusion testing of BFGI, using the zone diameter breakpoints published by Nagy et al. in 2015 [10], and compared the results of the two methods to each other.

## 2. Results

### 2.1. Distribution of Isolates

Table 1 summarizes the distribution by genus and/or species of the 224 anaerobic bacteria and the in-vitro resistance rates to the tested antibiotics determined by MIC gradient testing. All isolates included in the study were clinically relevant. From 224 isolates, 114 (50.9%) were Gram-positives, including *Finegoldia magna (n* = 31), *Peptoniphilus assacharolyticus* (*n* = 23), *Peptoniphilus* spp. (*n* = 13), *Peptostreptococcus anaerobius* (*n* = 8), *Parvimonas micra* (*n* = 8), *Actinomyces* spp. (*n =* 11), *Cutibacterium acnes* (*n* = 15), and *Cutibacterium avidum* (*n* = 5). Furthermore, 110 (49.1%) strains of the BFGI were tested. The most frequently isolated species among the BFGI was *Bacteroides fragilis* (*n* = 51), followed by *B. thetaiotaomicron* (*n* = 19), *B. ovatus* (*n* = 14), *B. vulgatus* (*n* = 11), *Parabacteroides distasonis* (*n* = 6), *B. uniformis* (*n* = 5), *B. caccae* (*n* = 2), and *B. stercoris* (*n* = 2).

### 2.2. Antibiotic Susceptibility (MIC)

Within the 114 Gram-positive anaerobic isolates, the highest resistance rates were found for clindamycin with 21.9% (25; 12 *F. magna*, 6 *P. assacharolyticus*, 2 *P. micra*, 4 *Actinomyces* spp., 1 *P. avidum*), followed by moxifloxacin with 16.7% (19; 12 *F. magna*, 5 *P. anaerobius* and 2 *Peptoniphilus* spp.). Low resistance levels were found for penicillin, amoxicillin/clavulanic acid, piperacillin/tazobactam (1 *P. anaerobius*), and for metronidazole (1 *F. magna*). Resistance rates for metronidazole of *C. acnes* and *C. avidum* were not counted, as most of these strains were naturally resistant to metronidazole [11]. No resistance was detected for imipenem, meropenem, and vancomycin.

Within the 110 BFGI, resistance rates were as follows (Table 1): 50.9% for clindamycin (56; 19 *B. fragilis*, 37 non-fragilis species), followed by moxifloxacin with 36.4% (40; 22 *B. fragilis*, 18 non-fragilis species). For piperacillin/tazobactam, a resistance rate of 10% (11; 1 *B. fragilis*, 10 non-fragilis species) was observed in contrast to amoxicillin/clavulanic acid with 7.3% (8; 4 *B. fragilis*, 4 *Bacteroides* non-fragilis isolates; BNFI). Low resistance rates were detected for metronidazole with 2.7% (3; all BNFI) and carbapenems with 1.8% (2; all *B. fragilis*). One *B. uniformis* and one *B. caccae* showed MIC values <0.5 mg/L to penicillin (0.25 and 0.032 mg/L, respectively), 98.2% (108) of BFGI were resistant to penicillin. Two *B. fragilis* isolates were classified as multi drug resistant (MDR). These two strains, isolated from wounds, were resistant to all tested beta-lactam antibiotics including the carbapenems. Additionally, one of the two isolates was resistant to clindamycin, and the other one to moxifloxacin.

### 2.3. Disc Diffusion Testing of BFGI

In 109 of 110 BFGI, susceptibility was additionally determined by disc diffusion. Results are depicted in Table 2 and Table 3. There were 130 discrepancies in 763 readings (17%) between results determined by disc diffusion compared to those determined by MIC testing. The number of very major errors (VME) was as follows: 50% for imipenem, 37.5% for amoxicillin/clavulanic acid, 7.1% for clindamycin, 10% for moxifloxacin, and 33.3% for metronidazole; there were no VME for piperacillin/tazobactam. The number of major errors (ME) was also high: 40.2% for piperacillin/tazobactam, 8.3% for imipenem, 16.7% for clindamycin, 3.6% for moxifloxacin, and 29.9% for metronidazole. The number of minor errors (mE) was 2.7% for amoxicillin/clavulanic acid, 10.9% for piperacillin/tazobactam, 0.9% for imipenem, and 13.6% for moxifloxacin. Inhibition zone diameters (mean ± standard deviation) for the B. fragilis ATCC 25285 control strain in parallel measurements on 8 different dates were as follows: amoxicillin/clavulanic acid 29.3 mm (±1.2), piperacillin/tazobactam 27.6 mm (±1.1), imipenem 31.6 mm (±1.7), clindamycin 28.8 mm (±1.9), moxifloxacin 27.5 mm (±1.4), and metronidazole 24.3 mm (±3.7).

## 3. Discussion

Infections due to anaerobic bacteria play an important role in clinical routine. A large study including 365,490 episodes of healthcare-associated infections found that *Bacteroides* spp. were under the 15 most common pathogens causing nosocomial infections [2]. Most infections, where anaerobic bacteria are involved, were treated empirically, mainly with betalactam antibiotics [5]. Due to worldwide increase in resistance rates, especially for *Bacteroides* spp., and extensive differences in the antimicrobial susceptibility patterns of anaerobic bacteria isolated within different countries, local surveillance studies on a regular basis are highly recommended [9,12].

In this study, we evaluated the antibiotic susceptibility profile of different Gram-positive anaerobic bacteria and BFGI isolated from human clinical specimens such as intra-abdominal infections, wounds, and abscesses. Furthermore, we performed disc diffusion testing of BFGI and compared the results to those determined by MIC gradient testing. The findings of this surveillance study present constantly low resistance levels for Gram-positive anaerobic bacteria in Austria. Remarkable resistance was only observed for clindamycin (21.9%) and moxifloxacin (16.7%). Based on our findings, betalactam antibiotics, vancomycin, and metronidazole remain useful drugs for empiric treatment of anaerobic Gram-positive infections. One exception is *Cutibacteria* spp. due to their natural resistance to metronidazole [11].

Within the BFGI, we are facing increasing resistance rates. In the year 2007, we found very low resistance rates (<1%) to amoxicillin/clavulanic acid, piperacillin/tazobactam, the carbapenems, and metronidazole [13]. Compared to this study, resistance levels increased for all tested antibiotics. Based on our findings, clindamycin and moxifloxacin showed remarkable resistance (50.9% and 36.4%, respectively). Consequently, they are not recommended for empiric treatment. The resistance rates for carbapenems are still low (0.9% for imipenem and 1.8% for meropenem), but there must be an increasing awareness of multidrug resistance among BFGI. Monitoring the resistance patterns of BFGI in routine laboratories by disc diffusion testing, which is much less expensive and easier to perform, would facilitate surveillance. In 2015, tentative zone diameter susceptibility breakpoints for six different antibiotics for disk diffusion testing of BFGI were published [10]. In this study, we compared results of disc diffusion testing to MIC values determined by gradient testing. The number of discrepancies between the two methods were very high, especially for piperacillin/tazobactam (47 discrepant results), moxifloxacin (21 discrepant results), and metronidazole (33 discrepancies). The number of VME and ME was also high for all tested antibiotics. Most of the isolates that showed discrepancies were close to the breakpoint.

This study has limitations. The results of disc diffusion testing were compared to MICs obtained by MIC gradient test method (ETEST^®^), not the agar dilution method, which would be the preferred technique, but, due to its labor-intensive and time-consuming character, is not suitable for routine diagnostic procedures. In contrast to the study from Nagy et al., bacterial suspension was prepared in Brucella broth, not in 0.85% saline or thioglycolate broth, and all discs were from a different company than those in the study from Nagy et al. (BioRad, France and Oxoid, UK versus Becton Dickinson, Heidelberg, Germany). Therefore, we cannot exclude the possibility that these differences would have led to these results. Furthermore, we had some problems with the metronidazole disc. The zone diameter of metronidazole of the *B. fragilis* ATCC 25285 control strain ranged from 20 to 28 mm within eight measurements. Therefore, we cannot ensure that results of metronidazole disc diffusion testing are reliable. As described before, this method may be used as a screening method [14]; we would suggest that all results close to the breakpoint are confirmed by a MIC method.

## 4. Materials and Methods

### 4.1. Isolate Collection

Clinical specimens from wounds, abscesses, ulcers, intra-abdominal, and urogenital infection (Table 4) were collected as part of the standard health care at the University Hospital Graz, Austria. Specimens were cultured on Schaedler agar with Vitamin K1 and 5% Sheep Blood and KV agar (Becton Dickinson, Germany) for 48 h in anaerobic atmosphere. Suspected anaerobe isolates were identified using Matrix-Assisted Laser Desorption/Ionization Time-Of-Flight Mass-Spectrometry (MALDI-TOF MS) (Vitek^®^ MS, bioMérieux, Marcy-l’Étoile, France). If there was no result, 16S rRNA Gene sequencing was performed for identification. Isolates were stored in Viabank™ tubes (MWE Medical Wire, Wiltshire, UK) at −80 °C at the Research & Diagnostic Institute of Hygiene, Microbiology and Environmental Medicine, Medical University of Graz, Austria.

### 4.2. Susceptibility Testing

In total, 224 non-duplicate anaerobic bacterial isolates were analysed. For susceptibility testing, isolates were thawed on Schaedler agar and sub-cultured once prior to inoculation. For MIC determination ETEST^®^ (bioMérieux, France) or MIC test strips (Liofilchem, Roseto degli Abruzzi, Italy) with the protocol for anaerobic bacteria were used. For both ETEST^®^ and disc diffusion, a bacterial suspension was prepared in Brucella broth (Becton Dickinson, Germany) to a density of McFarland 1. AST was performed on Brucella agar with Vitamin K1, 5% blood, and hemin (Becton Dickinson, Germany). The 1515-15-min rule of EUCAST was applied. The plates were incubated at 35 °C in an anaerobic atmosphere for 48 h. For Gram-positive anaerobic bacteria, MICs of the following agents were determined: penicillin, amoxicillin/clavulanic acid, piperacillin/tazobactam, imipenem, meropenem, moxifloxacin, vancomycin, clindamycin, and metronidazole. For the BFGI, MICs of the following agents were determined: amoxicillin/clavulanic acid, piperacillin/tazobactam, imipenem, meropenem, moxifloxacin, cefoxitin, clindamycin, and metronidazole. Interpretation was done according to EUCAST guidelines [15] for anaerobes, except for cefoxitin and moxifloxacin, where the CLSI guideline M11-A8 [16] was applied. Disk diffusion testing was performed for the same antimicrobial agents except for cefoxitin and meropenem. All discs (amoxicillin/clavulanic acid 20/10 µg, piperacillin/tazobactam 30/6 µg, imipenem 10 µg, moxifloxacin 5 µg, clindamycin 10 µg, and metronidazole 5 µg) were obtained from Becton Dickinson (Germany). For interpretation, the 2015 published zone diameter from Nagy et al. was used [10]: amoxicillin/clavulanic acid (≥15 mm), piperacillin/tazobactam (≥25 mm), imipenem (≥29 mm), clindamycin (≥25 mm), moxifloxacin (≥19 mm), and metronidazole (≥24 mm). All the measurements were carried out with the naked eye using a ruler, and all measurements were done by the same person. Zone diameters were read at 100% inhibition after 24 h of incubation. The proportion of VME (disc diffusion = S and reference method = R), ME (disc diffusion = R and reference method = S), and mE (disc diffusion = S or R and reference method = intermediate, I) between disc diffusion and MIC determination (reference method) was calculated [17]. *B. fragilis* ATCC 25285 was included as the quality control strain.

## 5. Conclusions

In conclusion, our data still show low resistance rates for betalactam antibiotics and metronidazole for anaerobic bacteria. Remarkable resistance was only observed for clindamycin and moxifloxacin. There should be awareness of increasing resistance rates, especially for *Bacteroides* spp. Therefore, disc diffusion testing, which can be performed easily in all microbiological laboratories, would be needed, but the method studied has potential for improvement. Surveillance studies on a regular basis are still recommended.

## Figures and Tables

**Table 1:** Species distribution and in vitro resistance rates determined by MIC.

**Antibiotic** **Resistance Breakpoint (mg/L)**	**P** **>0.5**	**AMC** **>8**	**TZP** **>16**	**IMP** **>4**	**MEM** **>8**	**CC** **>4**	**MOX** **>4**	**MTZ** **>4**	**VA** **>2**
	Resistance at breakpoint % (*n*)								
*Finegoldia magna* (31)	0	0	0	0	0	38.7 (12)	38.7 (12)	3.2 (1)	0
*Peptoniphilus assacharolyticus* (23)	0	0	0	0	0	26.1% (6)	0	0	0
*Peptoniphilus* spp. (13)	0	0	0	0	0	0	15.4 (2)	0	0
*Peptrostreptococcus anaerobius* (8)	12.5 (1)	12.5 (1)	12.5 (1)	0	0	0	62.5 (5)	0	0
*Parvimonas micra* (8)	0	0	0	0	0	25 (2)	0	0	0
*Actinomyces* spp. (11)	0	0	0	0	0	36.4 (4)	0	0	0
*Cutibacterium acnes* (15)	0	0	0	0	0	0	0	100 (15)	0
*Cutibacterium avidum* (5)	0	0	0	0	0	20 (1)	0	80 (4)	0
**Gram positive Anaerobes (114)**	**0.9 (1)**	**0.9 (1)**	**0.9 (1)**	**0**	**0**	**21.9 (25)**	**16.7 (19)**	0.9 (1) *	**0**
**Antibiotic** **Resistance Breakpoint (mg/L)**	**P** **>0.5**	**AMC** **>8**	**TZP** **>16**	**IMP** **>4**	**MEM** **>8**	**CC** **>4**	**MOX** **>4**	**MTZ** **>4**	**FOX** **>32**
	Resistance at breakpoint % (*n*)								
*Bacteroides fragilis* (51)	100 (51)	7.8 (4)	2 (1)	3.9 (2)	3.9 (2)	37.3 (19)	43.1 (22)	0	5.9 (3)
*Bacteroides thetaiotaomicron* (19)	100 (19)	10.5 (2)	21 (4)	0	0	84.2 (16)	47.4 (9)	0	52.6 (10)
*Bacteroides ovatus* (14)	100 (14)	0	0	0	0	64.3 (9)	28.6 (4)	7.1 (1)	28.6 (4)
*Bacteroides vulgatus* (11)	100 (11)	18.2 (2)	18.2 (2)	0	0	54.5 (6)	18.2 (2)	0	0
*Parabacteroides distasonis* (6)	100 (6)	0	66.6 (4)	0	0	66.6 (4)	16.7 (1)	33.3 (2)	33.3 (2)
*Bacteroides* spp.** (9)	77.8 (7)	0	0	0	0	22.2 (2)	22.2 (2)	0	0
**BFGI (110)**	**98.2 (108)**	**7.3 (8)**	**10 (11)**	**1.8 (2)**	**1.8 (2)**	**50.9 (56)**	**36.4 (40)**	**2.7 (3)**	**17.3 (19)**

P, penicillin; AMC, amoxicillin/clavulanic acid; TZP, piperacillin/tazobactam; IMP, imipenem; MEM, meropenem; CC, clindamycin; MOX, moxifloxacin; MTZ, metronidazole; VA, vancomycin; FOX, cefoxitin; * *Cutibacteria* spp. were excluded because of natural resistance to metronidazole; ** *Bacteroides uniformis* (5), *Bacteroides caccae* (2), *Bacteroides stercoris* (2).

**Table 2:** Results of susceptibility testing by disc diffusion for the Bacteroides fragilis group isolates (BFGI), breakpoints of zone diameters according to Nagy et al., 2015 [10].

Antibiotic	AMC		TZP		IMP		CC		MOX		MTZ	
Zone Diameter Breakpoints (mm)	<15	≥15	<25	≥25	<29	≥29	<25	≥25	<19	≥19	<24	≥24
*Bacteroides fragilis* (51)	1	50	10	41	2	49	18	33	23	28	6	45
*Bacteroides thetaiotaomicron* (19)	2	17	19	0	2	17	16	3	9	10	9	10
*Bacteroides ovatus* (14)	0	14	11	3	0	14	10	4	6	8	7	7
*Bacteroides vulgatus* (11)	2	9	10	1	0	11	8	3	2	9	2	9
*Parabacteroides distasonis* (6)	0	6	6	0	6	0	6	0	1	5	6	0
Bacteroides ssp.** (8)	0	8	2	6	0	8	2	6	0	8	4	4
BFGI (109) **	**5**	**104**	**58**	**51**	**10**	**99**	**60**	**49**	**41**	**68**	**34**	**75**

AMC, amoxicillin/clavulanic acid; TZP, piperacillin/tazobactam; IMP, imipenem; CC, clindamycin; MOX, moxifloxacin; MTZ, metronidazole; ** *B. uniformis* (4) no growth of 1 strain of *B. uniformis*, *B. caccae* (2), B. stercoris (2).

**Table 3:** Minor errors (mE), major errors (ME), and very major errors (VME) for disc diffusion versus MIC testing for *Bacteroides fragilis* group isolates (BFGI).

BFGI (109) *	AMC	TZP	IMP	CC	MOX	MTZ
**Discrepant results % (*n*)**	5.5% (6)	43.1% (47)	9.2% (10)	11.9% (13)	18.3% (20)	30.3% (33)
mE	2.8% (3)	11% (12)	0	n.a.	12.8% (14)	n.a.
ME	0	40.7% (35)	8.4% (9)	16.7% (9)	3.6% (2)	30.2% (32)
VME	37.5% (3)	0	50% (1)	7.3% (4)	10.3% (4)	33.3% (1)
**Total errors**	**45.8%**	**51.7%**	**58.4%**	**24%**	**26.7%**	**63.5%**

AMC, amoxicillin/clavulanic acid; TZP, piperacillin/tazobactam; IMP, imipenem; CC, clindamycin; MOX, moxifloxacin; MTZ, metronidazole; mE, minor error (disc diffusion = S or R and reference method = I), ME, major error (disc diffusion = R and reference method = S), VME, very major error (disc diffusion = S and reference method = R); n.a., not applicable, because no I interpretation; * 1 strain not readable in disc diffusion test.

**Table 4:** Sources of the species.

**Gram positive Anaerobes (114)**	***n***	**%**
Wounds/decubita/ulcer	53	46.5
Abscesses	21	18.4
Infection/abscesses in the oral cavity	14	12.3
Urogenital	12	10.5
Perianal abscesses/Sinus pilonidalis	12	10.5
Other	2	1.8
**BFGI (110)**	***n***	**%**
Wounds/decubita/ulcer	40	36.4
Intraabdominal infections/abscesses	32	29.1
Urogenital	18	16.4
Perianal abscesses/Sinus pilonidalis	10	9.1
Other	6	5.5
Abscesses	4	3.6
